# Hydroalcoholic extract and seed of *Foeniculum vulgare* improve folliculogenesis and total antioxidant capacity level in F1 female mice offspring

**DOI:** 10.1186/s12906-020-03083-3

**Published:** 2020-09-29

**Authors:** Fahimeh Pourjafari, Tahereh Haghpanah, Seyed Noreddin Nematollahi-Mahani, Fariba Pourjafari, Massood Ezzatabadipour

**Affiliations:** 1 Department of Anatomical Sciences, Faculty of Medicine, Kerman University of Medical Sciences, Kerman, Iran; 2 Department of Hematology and Medical Laboratory Sciences, Faculty of Allied Medicine, Kerman University of Medical Sciences, Kerman, Iran

**Keywords:** *Foeniculum vulgare*, Folliculogenesis, Total antioxidant capacity, First generation

## Abstract

**Background:**

*Foeniculum vulgare* (fennel) is traditionally suggested for the fertility improvement in Iranian lore due to its antioxidant and phytoestrogen compounds. The present study aimed to investigate the effects of fennel seed and its hydroalcoholic extract on the serum total antioxidant capacity (TAC) and folliculogenesis in offspring exposed to either of the treatments in utero and 56 days after birth (PND 56).

**Methods:**

Pregnant NMRI mice were randomly divided into 5 groups of 7: extract-treated groups received 500 and 1000 mg/kg/day fennel extract (FE), seed-treated groups received 500 and 1000 mg/kg/day fennel seed (FS), and the control group (CTL) received no treatment. The treatments started from pregnancy day 1 and continued until PND 56. Body and right ovary weights and ovary dimensions were recorded. Hematoxylin and eosin stained ovary sections were prepared to calculate the proportion of different follicles. The level of TAC in the serums was also measured by fluorescence recovery after photo bleaching.

**Results:**

A marked rise in the body and ovary weights of treated mice was observed compared to the CTL group. The mean number of primordial, primary, pre-antral, and pre-ovulatory follicles as well as corpus luteum size in the treated offspring was significantly higher compared to those of CTL offspring. The atretic follicle number was nonsignificantly lower in either of the treatment groups compared with that in the CTL. However, treatment of animals with 500 mg/kg FE showed a more pronounced effect. Animals in FS500, FE500 and FE1000 groups had a significantly higher level of serum TAC compared to the CTL group.

**Conclusions:**

Fennel extract and seed administration in pregnancy and lactation period improve offspring’s folliculogenesis. Higher level of TAC in the serum of offspring might have positively altered the folliculogenesis milieu.

## Background

Today’s world is full of environmental stressors including pollutants, radiations, chemicals and toxins which could alter cellular redox homeostasis and lead to excessive production of reactive oxygen species (ROS) which is normally compensated for the ROS attacks by antioxidant systems [1]. An imbalance between body antioxidant system and ROS production could negatively affect many body systems such as reproductive system [2, 3]. Oxidative stress is associated with a reduced follicle number and disturbed ovarian function, which may negatively influence women’s fertility [4]. One way to withstand this obstacle is the reinforcement of the antioxidant system by consumption of natural antioxidant compounds in the diet. The usage of herbal products with high concentration of antioxidant compounds could resist oxidative stress inducers and enhance fertility. Among the various compounds, phenolic substances such as flavonoids, phenolic acids, coumarins, tocopherols, and cinnamic acid derivatives are responsible for antioxidant properties frequently observed in several medicinal plants such as *Foeniculu vulgare Mill* (Apiaceae. Family); commonly known as fennel. Fennel is a biennial plant and is native to central Europe, the Mediterranean region, and Iran [5, 6]. Several studies have reported the improving effect of fennel on the function of the female reproductive system as well as its ameliorating effect on ovarian toxicity. These improving effects attribute have beend to its antioxidant compounds [4, 7]. Fennel is commonly used as seeds, oil, or in different types of extracts such as aqueous, alcoholic, acetonic, and hydro-alcoholic. Studies have shown the strong antioxidant activity of aqueous and ethanolic extracts of fennel in different antioxidant assessment methods compared to standard antioxidant solutions such as butylated hydroxyl anisole, butylated hydroxyl toluene, and alpha-tocopherol [8]. It is reported that in comparison to fennel seeds, its acetonic extract contains more phenolic compounds. In addition, its methanolic extract has 50% more flavonoid compounds compared to that of fennel seed, itself [9, 10]. Consumption of ethanolic extract of fennel (100 and 200 mg/kg) could reduce the level of oxidants and malondialdehyde (MDA) in mouse serum [11, 12] and also induce folliculogenesis through increasing the number of growing follicles in female mice due to its estrogenic and antioxidant compounds [4]. Furthermore, the administration of fennel oil (1 mg/kg body weight) could exhibit protective effects on folliculogenesis against cyclophosphamide-induced reproductive toxicity in female rats, which might be due to the increase of antioxidant defense systems [13].

It is well established that mother’s diet during pregnancy and lactation affects pups’ reproductive system development and function. In our recent study [14], we observed that the consumption of hydroalcoholic extract of fennel (500 mg/kg) during pre- and post-natal periods improve offspring folliculogenesis and reduce apoptotic follicles. Whether taking higher doses of this extract has the same effects or whether the seeds of fennel can yield similar or better results remains to be investigated which is the goal of the present study in which the effect of different doses of fennel (extract & seed) on offspring folliculogenesis based on the TAC level in the serum.

## Methods

### Plant material and hydroalcoholic extract preparation

Fennel (*Foeniculum vulgare*) was purchased from local market and authenticated by Department of Pharmacognosy, Kerman University of Medical Sciences, Kerman, Iran (KMU). The seed of fennel (Voucher number: KF1466) was grinded and passed through a sieve (mesh 300). The powder (100 g) was extracted using warm maceration method with 80% ethanol for 72 h. The extract was concentrated in a vacuum condition, dried in oven at 40 °C for 48 h and held at − 20 °C until use [15].

### Animals and study design

Twenty female and ten male NMRI mice, weighing 25–30 g (6–8 weeks old), were purchased from the animal house affiliated with Afzalipour School of Medicine, Kerman, Iran.

The animals were housed in cages containing wood fiber bedding (4 mice/ cage) under well-controlled conditions of temperature (21 ± 2 °C) and light/dark cycle (12/12 h) with free access to water and usual rodent food. The experimental protocol was approved by the institutional ethics committee of Kerman University of Medical Sciences (approval number: IR.KMU.REC.1397.148).

For mating, two female and one male mouse were placed in each cage. Next morning, the presence of a vaginal plaque was considered as the first day of pregnancy. Pregnant animals (*n* = 20) were randomly classified in five groups as follows: the control (CTL) group received usual rodent food with no additional treatment. Animals in the extract-treated groups received 500 mg/kg/day (FE500) [16] and 1000 mg/kg/day (FE1000) [17] hydroalcoholic extract of fennel. While, the animals in seed-treated groups received 500 mg/kg/day (FS500) and 1000 mg/kg/day (FS1000) fennel seed. The extracts were mixed with the animals’ diet weekly. In order to ascertain that each animal received the exact dose of extract, the daily food intake of some mice was measured by subtracting the weight of the food at 8 a.m. and the weight of the food at next 8 a.m. for 2 weeks to estimate the daily food intake. The extract was then added to the rodent food according to the body weight (kg).

The treatments were performed throughout pregnancy and lactation. After weaning, the female offspring were separated, kept in home cage and the treatments were continued, until postnatal day 56 (PND 56) [18]. Totally, thirty-five female pups (8 weeks old, 7 in each group) were used. Each group was included seven mice. This sample size was set following an extensive literature review and available methods such as E and power analysis methods.

### Ovary collection

At PND56, pups were weighed and euthanized by cervical dislocation. The right ovaries were removed, weighted by digital scale and the large and small diameters were measured by a digital caliper and were fixed in 10% (v/v) buffered formalin for 24 h.

### Histological study of the ovaries

Specimens were dehydrated with increasing concentration of alcohol (50, 70, 80, 90 and 100%, respectively), embedded in paraffin wax and serially sectioned at 5 μm thickness by a rotary microtome (Did sabz, Iran). Ten sections were selected with 50 μm interval, the samples were stained by hematoxylin and eosin and were studied under a light microscope (Olympus IX51, Japan).

### Classification of ovarian follicles

Follicles were classified into different types including primordial, primary, secondary, pre antral, antral, pre ovulatory and atretic follicles. The number of different follicles and also corpus luteum were assessed in each section [19].

### Blood collection

Blood samples were collected from left ventricle under anesthesia (combination of ketamine (5–10 mg/kg) and xylazine (5 mg/kg) (mixture of ketamine as a dissociative agent and xylazine as an α2-adrenoreceptor agonist is one of well-established general anesthetic modalities which commonly used in laboratory mice). The collected blood was then centrifuged at 2000–2500 rpm for 10 min and the serum was carefully removed and kept at − 20 °C until biochemical analysis.

### Assay of serum total antioxidant capacity (TAC) level

Serum TAC level was determined by ferric ions reducing antioxidant power (FRAP) method according to TAC commercial kit (Navand salamat Company, Urmia, Iran). In this method, due to the presence of antioxidants, a reduction of ferric to ferrous ion at low pH occurs and a colored ferrous-tripyridyltriazine complex is formed which can be measured spectro-photometrically at 593 nm [20].

### Statistical analysis

The data were analyzed by version 16 SPSS software for Windows. The normality of the data was tested by using the One-sample Kolmogorov-Smirnov test. The parametric data were analyzed by one-way analysis of variance (ANOVA) followed by Tukey’s post hoc and non-parametric data were analyzed by the Kruskal-Wallis test. The values were presented as mean ± standard error of the mean (SEM). Significance was established when *p* < 0.05.

## Results

### Effect of fennel extract and seed on body and ovary weights

The effect of the seed and hydroalcoholic extract of fennel on the body and ovary weights is represented in Table 1. Female offspring mice treated with seed or extract at any given doses (*n* = 7) showed a significant weight gain compared to that of the control mice (*p* < 0.01).

**Table 1:** Mice body, ovary weight and diameters in the different groups (*n* = 7)

Parameters	Groups				
	CTL	FS500	FS1000	FE500	FE1000
**Mice weight (g)**	18.82 ± 0.69	24.47 ± 0.47^a**^	24.84 ± 0.62^a**^	25.17 ± 0.54^a**^	25.1 ± 0.52^a**^
**Ovary weight (mg)**	2 ± 0.3	3.2 ± 0.6	3.4 ± 0.2^a**^	5 ± 0.6^a**bc*^	4.5 ± 0.5^a**^
**Large diameter of ovary (mm)**	2.04 ± 0.08	2.01 ± 0.07	1.9 ± 0.22	2.11 ± 0.21	1.16 ± 0.08^abd**c*^
**Small diameter of ovary (mm)**	1.65 ± 0.11	1.37 ± 0.08	1.8 ± 0.22	1.86 ± 0.24	2.22 ± 0.08^a**^

The values are expressed as mean ± SEM. The values are comparable in the same row

^a^: a significant difference compared to the CTL group; ^b^: a significant difference compared to the SF500 group; ^c^: a significant difference compared to the SF1000 group; ^d^: a significant difference compared to the EF500 group; * and ** were shown *p* < 0.05 and *p* < 0.01, respectively

The weight of the ovary was higher in the treated pups with a statistically significant difference in the FS1000, FE500 and FE1000 compared to the control group (*p* < 0.01). It is noteworthy that the ovary weight in the FE500 group was highest with a significant difference compared to either of seed-treated groups; FS500 and FS1000 (*p* < 0.05).

### Effect of fennel extract and seed on the ovary diameters

As shown in Table 1, daily intake of hydroalcoholic extract of fennel (1000 mg/kg) significantly decreased large diameter of ovary compared to other experimental groups (CTL, SF500 and FS500; *p* < 0.01, FS1000; *p* < 0.05). This dose of extract significantly increased small diameter as compared with that of the control group (*p* < 0.01).

### Effect of fennel extract and seed on pups folliculogenesis

**Fig. 1:**
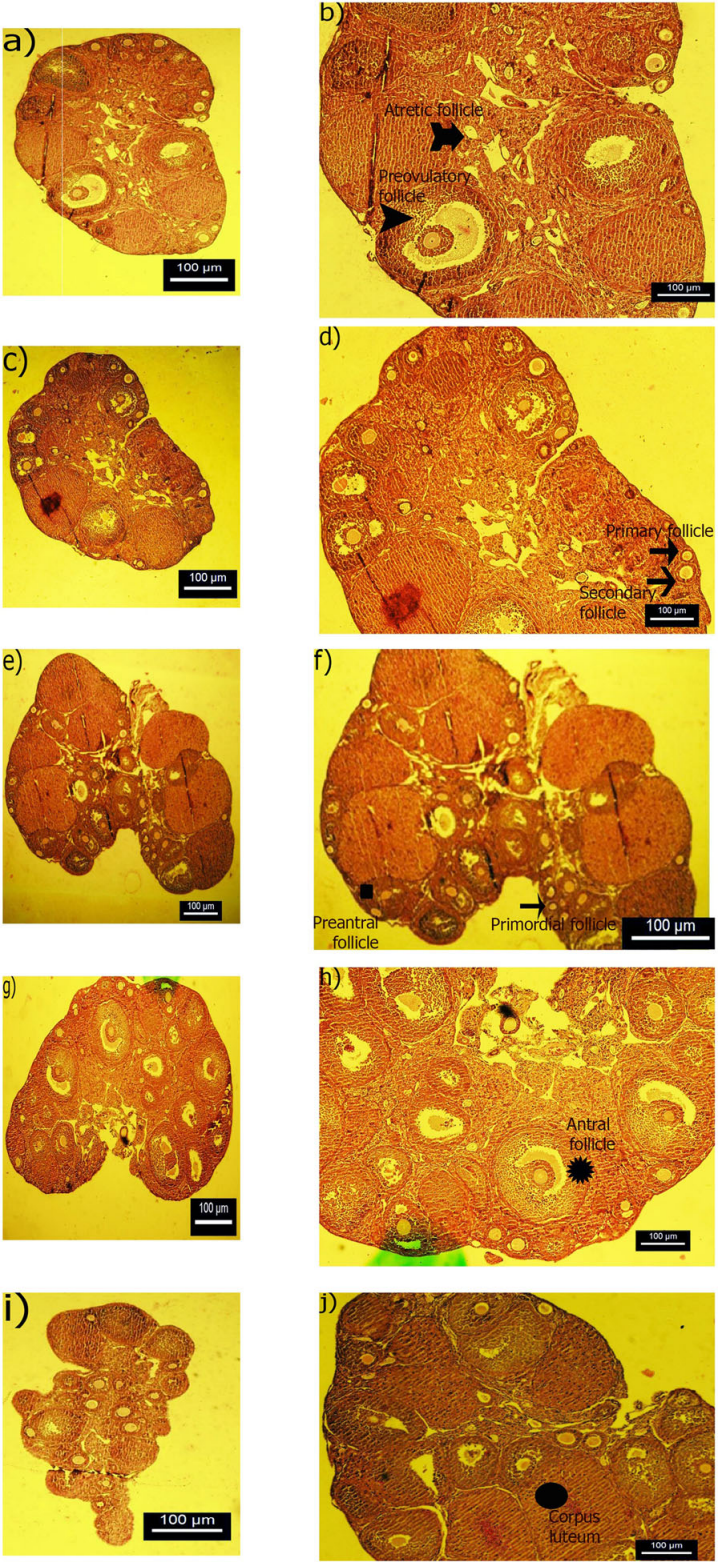
Photomicrographs of representative ovarian sections stained with Hematoxylin & Eosin in the different experimental groups. The control group (**a** & **b**: 40× and 100× magnification, respectively); the group treated with 500 mg/kg fennel seed extract (**c** & **d**: 40× and 100× magnification, respectively); the group treated with 1000 mg/kg fennel seed extract (**e** & **f**: 40× and 100× magnification, respectively); the group treated with 500 mg/kg fennel hydroalcoholic extract (**g** & **h**: 40× and 100× magnification, respectively); the group treated with 1000 mg/kg fennel hydroalcoholic extract (**i** & **j**: 40× and 100× magnification, respectively). Different follicles are shown in this figure

**Fig. 2:**
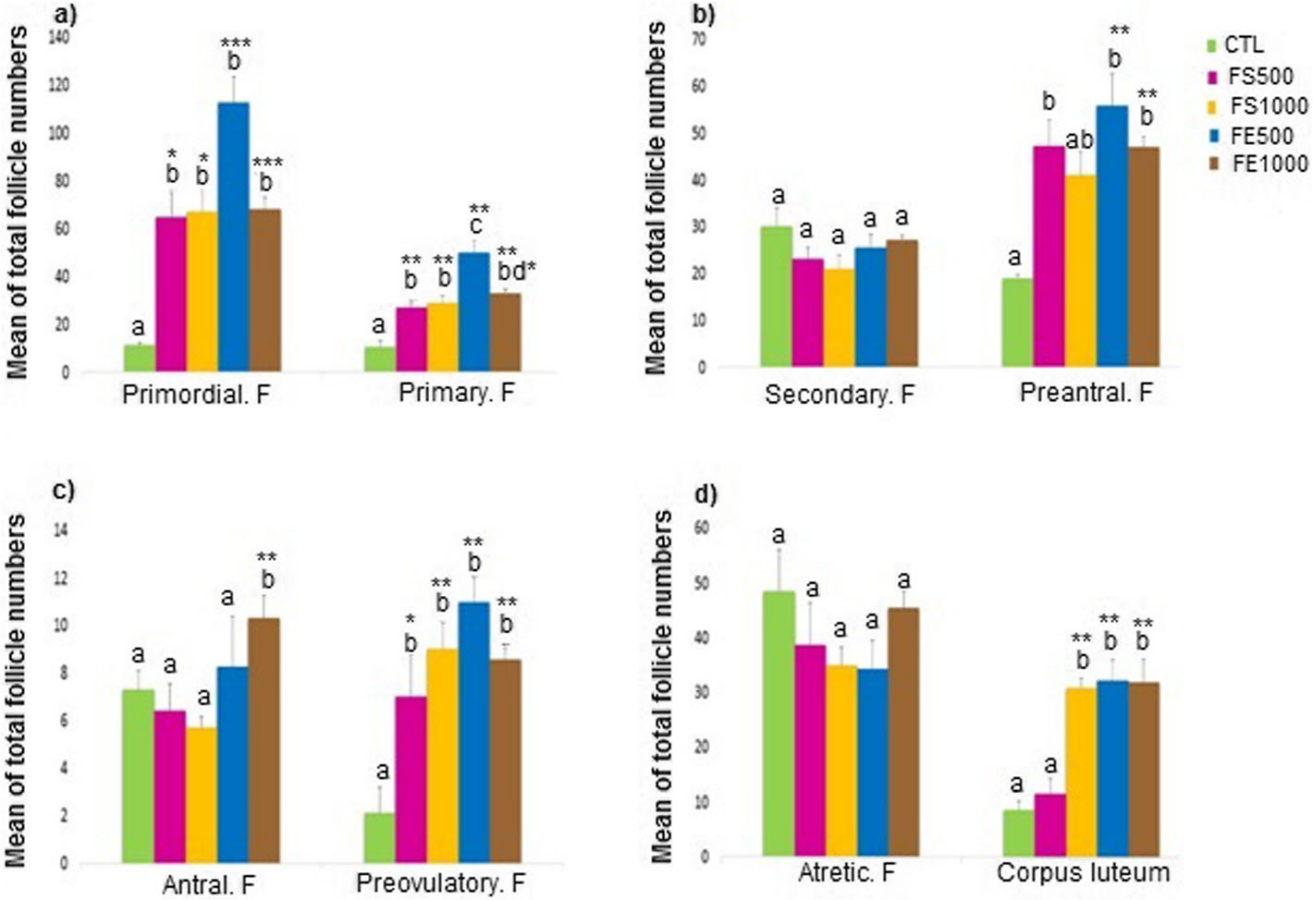
Distribution of different follicles in the experimental groups. The mean number of different follicles: primordial **&** primary (**a**), secondary & pre antral (**b**), antral & pre ovulatory (**c**) and atretic follicles & corpus luteum (**d**) are shown in the different groups. The same letters above the bar indicate no significant difference (*p* > 0.05). The different letters indicate a significant difference compared to the control group. Data are expressed as mean ± SEM.* *p* < 0.05, **p < 0.01 and ***p < 0.001

The different ovarian follicles were observed in all treated animals (Fig. 1). As shown in Fig. 2, the evaluation of histological sections of ovaries in the different groups revealed that there was a significant increase in the mean number of primordial follicles (Fig. 2 a) in either doses of the seed- treated mice (*p* < 0.05) and also either doses of extract-treated mice (*p* < 0.001) when compared to the control animals. The mean number of primary follicles had also a significant increase in all experimental groups (*p* < 0.01) compared to the control group. However, a significant higher number of primary follicles was observed in the fennel extract-treated mice at dose of 500 mg/kg compared with that in the other treated mice (*p* < 0.01 versus FS500 and *p* < 0.05 versus FS1000 and FE1000 groups) (Fig. 2a). There was no significant difference among the studied groups based on the secondary follicle number. Treatment with fennel, either its seed or extract, increased the mean number of pre-antral follicle (*p* < 0.01) (Fig. 2b). A significant high number of antral follicles was observed in FE1000- treated ovaries compared to other studied groups (*p* < 0.01). In addition, statistical analyses revealed a marked increase in the number of pre-ovulatory follicles in the ovaries of all treated mice when compared to the control mice (FS500; *p* < 0.05 and other groups; *p* < 0.01) (Fig. 2c). No significant difference was observed in the number of atretic follicle among the various groups. In the animals treated with fennel extract (500 and 1000 mg/kg) and fennel seed (1000 mg/kg), the number of corpus luteum increased significantly in comparison with the control and FS500 groups (*p* < 0.01) (Fig. 2d).

### Effect of fennel extract and seed on the serum TAC levels

**Fig. 3:**
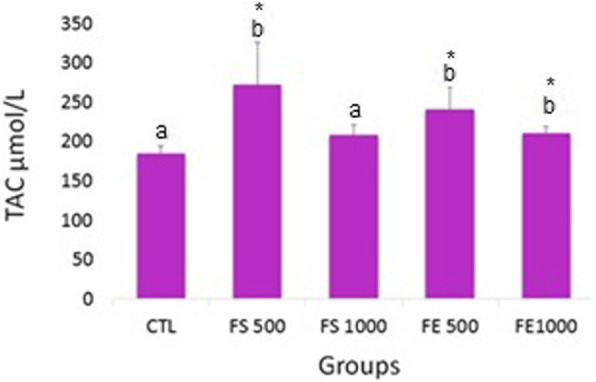
The administration effect of fennel either hydroalcoholic extract or its seed on the serum TAC concentration in the experimental groups. The same letter above the bar indicates no significant difference (*p* > 0.05). The different letter indicates a significant difference compared to the control group. *p* < 0.05 was considered as a significant level and shown*. Data are expressed as mean ± SEM (*n* = 7 for each group)

The level of serum TAC was significantly higher in the FS500, FE500 and FE1000 groups compared with that of the control group (*p* < 0.05) (Fig. 3).

## Discussion

The findings of the present study indicate that exposure of the female offspring mice to fennel hydroalcoholic extract or its seed during in utero development, breastfeeding and up to PND 56 could increase the body and ovary weights, change folliculogenesis pattern and increase serum TAC.

A marked rise in the average body weight was observed in all treated offspring. Exposure of pups to estrogenic drugs and numerous natural estrogenic substances such as phytoestrogen can stimulate adipogenic differentiation of stem cells, leading in turn to obesity, postnatally [21, 22]. Nikaido and co-workers reported that maternal exposure to phytoestrogens such as genistein, resveratrol, and zearalenone could increase body weight in the F1 female offspring compared with untreated mice [23]. Dietary intake of a diet rich in phytoestrogens structurally and functionally similar to endogenous estrogen could synergize the estrogen effect on body weight probably via reduction of metabolism and lipoprotein lipase activity in adipose tissues [21]. However, this effect depends on the dose and timing of exposure. As Nazari and co-workers reported that consumption of different concentration of fennel extract (75, 100 and 125 ml/ g diet) can increase weight gain and fertility rate in fish. They attributed these effects to phytoestrogen compounds in fennel [24]. A strong relationship between estrogen level and pituitary growth hormone secretion has been reported, as enhancing estrogen level following phytoestrogen intake may regulate body metabolism, growth, composition, weight and development in male and female [25]. This argument has been confirmed by a study showing an increased uterus and ovary weights following estradiol treatment [26]. All of the above-mentioned reports could be plausible mechanisms for increasing of the body weight following prenatally and postnatally exposure to fennel extract or its seed; rich in phytoestrogens.

Moreover, the ovary weight increased following feeding of the animals with either fennel extract or its seed; although the fennel extract showed a more pronounced effect. This ovarian weight gain may be explained by an increase in the number of growing follicles in the ovaries of treated mice. In line with our data, a previous study has reported an increase in the weight of the mammary glands, uterine tubules, ovaries, endometrium, myometrium, and vagina in female rats that received aqueous extract of fennel (150 mg/kg). They suggested that their results may be related to the estrogenic effect of fennel on genital organs that consequently leads to an increase of the ovary weight [27]. Hassanpour and colleagues showed that the fennel consumption could increase the ovary weight in mice exposed to cyclophosphamide due to fennel’s estrogenic properties [28]. Interestingly, in our study, a marked change in the ovary diameters was observed in the FE1000 group as the ovarian large diameter decreased significantly and its small diameter increased compared to the other groups. It appears that a change in the three-dimensional structure of the ovary with unknown reason has occurred as ovary shape changed from an oval to a circular form.

Estimation of the ovarian follicles number is usually performed to evaluate fertility potential [29]. Folliculogenesis is a complex process of growth and development of recruited primordial follicles, finally forming an ovulatory follicle, which has a potential for ovulation of the intra-follicular oocyte and formation of the corpus luteum. This pool of primordial follicles arises from the migration of primordial germ cells to genital ridge, and their growth and differentiation to oogonia and their supporting cells (follicular cells). Several reports have suggested that maternal lifestyle during key times of the ovarian development and primordial follicle formation in intrauterine developing fetuses may affect oogonia propagation and consequently, follicle numbers after puberty. One of the crucial factors in life style is diet. The mother’s diet can affect the offspring ovarian reserve. In our recent study, we observed that administration of hydroalcoholic extract of fennel (500 mg/kg) during pregnancy and lactation could improve folliculogenesis as well as ovarian reserve by decreasing the apoptosis rate in the ovarian follicles [14]. The results of the present study revealed that while the consumption of fennel in either forms (hydro-alcoholic and seed) and doses (500 and 1000 mg/kg) could have positive effects on offspring folliculogenesis, however the hydroalcoholic extract of fennel exert a pronounced effect on folliculogenesis when compared to fennel seed. The stronger effect of the hydroalcoholic extract of fennel in comparison to its seed may be explained by the high concentration of antioxidants in hydroalcoholic extract rather than its seed. This argument has been confirmed as the different effect of the extract and seed was also reported by De Nigris and colleagues [30]. They showed that supplementation of an atherogenic diet with pomegranate fruit extract can exert more beneficial effects on vascular function and inflammation in obese rats rather than pomegranate seed juice due to the abundance of polyphenolic antioxidants. In line with the positive effects of fennel on folliculogenesis, Khazaei et al. showed that intake of alcoholic extract of fennel (100 and 200 mg/kg) could markedly increase the number of graafian, antral, and multi-laminar follicles [4], which could lead to enhanced female infertility treatment [31]. The fennel extract could be utilized as a potent treatment to protect female fertility against ovarian toxicity caused by cyclophosphamide- and cadmium, via elevation of the serum levels of sex hormones and the number of ovarian follicles [28, 32]. However, the fennel effect depends on the dose of exposure. Isoflavone is one of the antioxidant compounds in fennel that its dose of exposure could significantly affect its function. A previous study has shown that prenatal and postnatal exposure to isoflavone; in higher doses in comparison with the its normal level, could alter the development of the female reproductive system in mice as well as early puberty, formation of multi-oocyte follicles and also alterations in the expression of estrogen receptors in the vagina and ovary through an estrogenic effect [33].

In ovaries of healthy women, more than 99% of follicles undergo destructive alterations known as atresia. Apoptosis or programmed cell death is the molecular mechanism underlying follicle atresia [34]. Since higher apoptosis rate could dramatically decrease the follicle numbers leading to a decrease in the women’s fertility, thus evaluation of the atretic follicles number is utilized as a beneficial model to understand apoptosis rate in ovarian tissue. In the present study, in line with other studies, fennel consumption decreased non-significantly the atretic follicle number in the treated groups compared to that of control animals. Several factors such as estrogen could serve as follicle survival and anti-apoptotic factors [35]. This sex hormone via estrogen receptor could lead to a reduction of Fas-ligand activity [36]. Because the phytoestrogens, estrogens-derived from plants, mimic the estrogen structure and functions, our data may be justified by the anti-apoptotic effect of fennel as a phytoestrogen- rich plant on the survival of the ovarian follicles in treated mice. In contrast to our data, a previous report determined that adult Sprague-Dawley rat offspring exposed to phytoestrogen daidzein during in utero development and neonatal period had a marked reduction in the height of ovarian surface epithelial cells, and alteration in folliculogenesis included an increase in follicular atresia and a reduction in the secondary and tertiary follicles number as well as cyst formation. Another observation was an elevated prevalence of a slightly prolonged estrus phase [37]. Although, the effect of fennel exposure on estrus cyclicity was not recorded in the present study, but there are some studies that have examined the effect of fennel exposure during in utero development and lactation periods on estrous cycle [38, 39].

Studies have shown that free radicals such as ROS, and environmental factors can cause DNA damage in the oocyte. A remarkable level of DNA damage may cause genomic abnormalities such as chromosomal breakages and mutations that lead to cell death and follicle atresia [40, 41]. A rise of TAC in the body could prevent the deleterious effect of oxidative stress on health. Several reports have shown a powerful antioxidant property in fennel due to the presence of antioxidant compounds such as isoflavenoid, phenolic acid and tocopherol. These antioxidants could protect the reproductive system via their free-radical scavenging activity and lipid oxidation inhibition [42]. This is a potential mechanism for the beneficial effects of fennel on facilitation of cell growth, prevention of follicular atresia and thus improving folliculogenesis [43]. According to the results of this study, TAC level was elevated in serum of the female offspring in all treated groups (seed and extract) when compared with that of the control animals. In line with our data, another study showed a significant increase of the TAC level in treated mice with 100 and 200 mg/kg doses of the fennel seed extract compared to the control mice. Also, a marked decrease in MDA level, an indicator of oxidative stress, was observed following administration of fennel as antioxidant in sperm cryopreservation [44]. Administration of fennel oil to cyclophosphamide-exposed animals caused an increase of antioxidant enzymes; superoxide dismutase and glutathione levels as well as a reduction of caspase3 expression level (apoptotic marker) in testis and liver tissues [13]. We did not measure the level of different antioxidants other than TAC in the serum of pups, which needs further investigation before being suggested in humans.

## Conclusion

In conclusion, the results of the present study determined an improvement in the ovarian folliculogenesis of F1 female offspring following the consumption of either fennel hydroalcoholic extract or its seed, although the hydroalcoholic extract showed a better effect in comparison to its seed. This may be a result of the elevated antioxidant capacity level and also survival and anti-apoptotic factors such as estrogen in the folliculogenesis milieu of the offspring’s ovaries. Overall, fennel extract or its seed can be used as an antioxidant component for improvement of female fertility.

## Data Availability

The datasets used and/or analyzed during the current study are available from the corresponding author on reasonable request.

## Abbreviations

TAC: Total antioxidant capacity
PND: Postnatal day
NMRI: Naval Medical Research Institute
FE: Fennel extract
FS: Fennel seed
CTL: Control
ROS: Reactive oxygen species
MDA: Malondealdehyde
KMU: Kerman University of Medical Science
FRAP: Ferric ions reducing antioxidant power
FS: *Foniculum vulgare* seed
